# Loss of miRNA-Mediated VEGFA Regulation by SNP-Induced Impairment: A Bioinformatic Analysis in Diabetic Complications

**DOI:** 10.3390/biomedicines13051192

**Published:** 2025-05-14

**Authors:** Raquel Freitas, Stela Felipe, Christina Pacheco, Emmanuelle Faria, Jonathan Martins, Jefferson Fortes, Denner Silva, Paulo Oliveira, Vania Ceccatto

**Affiliations:** 1 Laboratory of Biochemistry and Molecular Biology of UECE—LABIEX, Superior Institute of Biomedical Science—ISCB, State University of Ceará—UECE, Silas Munguba Avenue, 1700, Fortaleza 60714-903, CE, Brazil; stela.felipe@uece.br (S.F.); emmanuelle.santos@aluno.uece.br (E.F.); jonathan.elias@aluno.uece.br (J.M.); denner.silvino@aluno.uece.br (D.S.); paulo.elesson@aluno.uece.br (P.O.); vania.ceccatto@uece.br (V.C.); 2 Departamento de Biologia Celular e Molecular, Federal University of Paraíba—UFPB, João Pessoa 58051-900, PB, Brazil; christina.pacheco@academico.ufpb.br

**Keywords:** miRNAs, single nucleotide polymorphisms (SNPs), diabetic complications, vascular endothelial growth factor A (VEGFA)

## Abstract

**Background/Objectives**: MicroRNAs (miRNAs) are molecules involved in biological regulation processes, including type 2 diabetes and its complications development. Single nucleotide polymorphisms (SNPs) can alter miRNA mechanisms, resulting in loss or gain effects. VEGFA is recognized for its role in angiogenesis. However, its overexpression can lead to deleterious effects, such as disorganized and inefficient vasculature. Under hyperglycemic conditions, VEGFA expression seems to increase, which may contribute to the development of microvascular and macrovascular diabetic complications. Several miRNAs are associated with VEGFA regulation and seem to act in the prevention of dysregulated expression. This study aimed to investigate SNPs in miRNA regions related to the loss effect in VEGFA regulation, examining their frequency and potential physiological effects in the development of diabetic complications. **Methods**: VEGFA-targeting miRNAs were identified using the R package multimiR, with validated and predicted results. Tissue expression analysis and SNP search were data-mined with Python 3 for miRNASNP-v3 SNP raw databases. Allele frequencies were obtained from dbSNP. The miRNA–mRNA interaction comparison was obtained in the miRmap tool through Python 3. MalaCards were used to infer physiological disease association. **Results**: The variant rs371699284 was selected in hsa-miR-654-3p among 103 potential VEGFA-targeting miRNAs. This selected SNP demonstrated promising results in bioinformatics predictions, tissue-specific expression, and population frequency, highlighting its potential role in miRNA regulation and the resulting loss in VEGFA-silencing efficiency. **Conclusions**: Our findings suggest that carriers of rs1238947970 may increase susceptibility to diabetic microvascular and macrovascular complications. Furthermore, in vitro and in silico studies are necessary to better understand these processes.

## 1. Introduction

MicroRNAs (miRNA) are 17–22-nucleotide non-coding molecules that regulate various biological mechanisms through post-transcriptional processes. These small RNAs function by recognizing specific messenger RNA (mRNA) sequences, forming a silencing complex with endonucleases, which leads to mRNA degradation or translation repression [1].

The biogenesis of these RNAs can follow canonical or noncanonical paths, where single-nucleotide polymorphisms (SNPs) can spontaneously arise and alter binding affinity for mRNAs [2,3,4]. These sequence variations can result in a gain or loss of function in the miRNA silencing mechanism [5]. Nevertheless, the regulatory change mediated by SNPs in miRNAs can predispose carriers to these effects [6].

MiRNA expression profiles are recognized in several disorders and are considered strong biomarker candidates for diagnosis and prognosis in various conditions, including metabolic ones, such as diabetes mellitus [7,8]. Type 2 diabetes mellitus is a disorder affecting glucose and insulin mechanisms, which can lead to severe impacts on micro- and macrovascularization, potentially contributing to the development of diabetic complications [9,10,11].

VEGFA is essential for vascular growth and development, acting as a primary promoter of angiogenesis [12,13]. In insulin-resistant conditions, VEGFA expression appears to be elevated in the circulation, possibly due to the impacts of diabetes and inflammation. This pattern is also observed in tissues affected by diabetic complications. In this context, increases in VEGFA expression are linked to the development of angiogenesis and negative consequences, such as premature and disorganized vascular growth [14,15,16,17].

miRNAs are recognized as regulators of the VEGFA gene in the development of diabetes, potentially linking them to the prevention of vascular disorders [18]. SNPs in miRNA regions can influence their binding to target mRNAs, resulting in the loss of target silencing effects and subsequent overexpression [19]. These variants in miRNAs that target VEGFA may predispose carriers to diabetic complications mediated by gene dysregulation. In this context, our study aimed to investigate SNPs in miRNA regions targeting VEGFA that impair the silencing mechanism, describe the population frequency affected by these alleles, and characterize the physiological effects under the conditions of diabetes complications.

## 2. Materials and Methods

### 2.1. miRNA VEGFA Target

The evaluation of miRNAs targeting VEGFA was conducted using the MultimiR Package version 1.30.0 within the R environment version 4.4.2. The results were obtained according to the standard configurations of tools for miRNA prediction and validation databases [20]. Data from miRNA prediction and validation were compared to verify the concordance of the results.

### 2.2. miRNA Expression Profile in Diabetes-Related Tissues

The raw data standard tissue mean expression from the miRNA TissueAtlas 2025 version 3 (https://ccb-compute2.cs.uni-saarland.de/mirnatissueatlas_2025, accessed on 11 February 2025) was used to profile miRNA tissue expression. The selected tissues were artery, brain, cornea, heart, kidney, nerve, and vein under healthy conditions (the only available data in the database) [21]. These tissues were chosen as a reference for understanding potential dysregulation in disease contexts. Data mining was performed using Python Version 3 with the Pandas library 2.2.3 for data manipulation and aggregation [22,23]. Expression values were grouped by tissue type, and mean RPMM values were retained.

### 2.3. miRNA SNPs Associated with VEGF Regulation Alteration

The miRNASNP-v3 (https://guolab.wchscu.cn/miRNASNP#!/, accessed on 12 February 2025) raw database was consulted for SNPs in the top 10 miRNA-achieving regions with loss of function in VEGFA regulation [24]. Data mining was performed using the Python environment version 3, along with the Pandas version 2.2.3 library and the NumPy version 2.2.4 package [22,23,25].

The DbSNP Build 157 (https://www.ncbi.nlm.nih.gov/snp, accessed on 17 February 2025) database was accessed and manually curated for the miRNA SNP allele changes and frequency distribution in the global population. Thus, the most frequent allele and its continental representation were selected based on the tool-available studies [26]. Fisher’s exact test was applied using estimated allele counts derived from reported frequencies in genomic databases. The Bonferroni test was conducted to correct for multiple comparisons. Confidence intervals of 95% were utilized. Statistical significance was set at *p* < 0.05, following standard practice in population-based genomic studies [27]. The statistical evaluations were performed in Python environment version 3, along with the Pandas version 2.2.3 library, NumPy version 2.2.4 package, Matplotlib version 3.8.4, and Seaborn version 0.13.2 [22,23,25,28,29].

### 2.4. miRNA–mRNA Interaction

The miRNASNP-v3 database (https://guolab.wchscu.cn/miRNASNP#!/, accessed on 24 February 2025) was consulted to assess the SNP variation impact on miRNA interactions with VEGFA and gene 3′UTR binding locations [26]. The miRmap version 1.2.0 open-source package was utilized within a Python version 3 environment to evaluate the loss effects between the wild-type sequence and the mutant, considering thermodynamic aspects like free energy, probabilistic features, and sequence-based features, which were regarded as threshold scores ≥ 80 [30].

### 2.5. Evaluation of Physiological Implications

The Malacards version 5.24 (https://www.malacards.org/, accessed on 24 February 2025) tool was consulted to investigate the association between hsa-miR-654-3p and VEGFA in disease literature data, considering the tool’s algorithm for the association score and search score [31].

## 3. Results

### 3.1. microRNA Predicted and Validated for VEGFA Regulation

A total of 1807 interactions were identified with miRNAs–VEGFA through the MultiMiR package evaluation. From these, 437 miRNAs were identified, with 263 described in two or more tools as VEGFA regulators (Table S1).

**Figure 1:**
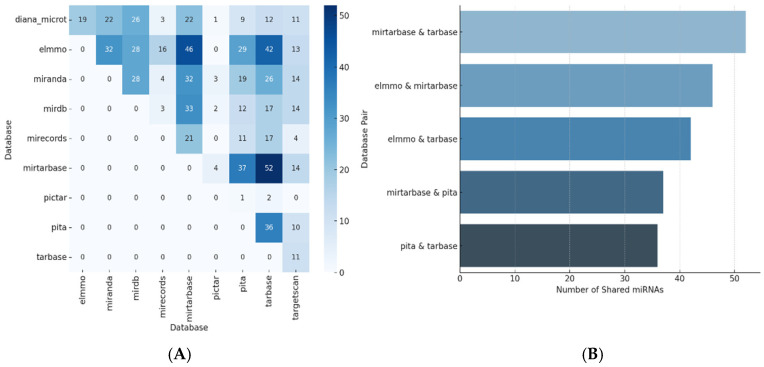
Results consonance through the prediction and validation of miRNA databases. (**A**) Shared miRNA results across databases. (**B**) Top 5 database interactions based on shared miRNA predictions.

The databases integrated by the MultimiR evaluation demonstrated consistency between predicted and validated miRNA results (Figure 1A and Table S2). Among the analyzed databases, several shared results; 24 interactions yielded the same miRNA results for at least one database, while 22 interactions identified no miRNAs.

The comparison of the top five database interactions (Figure 1B) highlighted values from over 30 miRNAs identified across databases. The highest levels of database consistency were shown by miRTarbase and Tarbase, which shared over 50 miRNAs over two validation databases.

**Figure 2:**
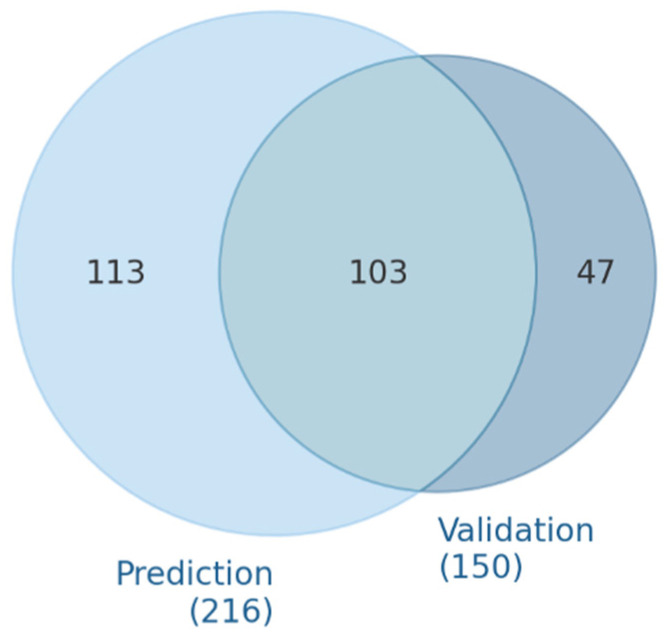
Overlap of predicted and experimentally validated miRNAs targeting VEGFA.

The prediction evaluation identified 216 miRNAs targeting VEGFA, while 150 experimentally validated gene–miRNA interactions were noted (Table S3). From these evaluations, 103 miRNAs were both predicted and validated (Figure 2).

### 3.2. miRNA Expression Profiles in Diabetes-Related Tissues

The analysis of 103 identified miRNAs revealed that many of these molecules exhibited expression patterns in organs commonly affected by diabetic complications (Table S4). However, they showed selectivity for certain tissues; hsa-mir-3941 exhibited the highest mean expression between the nerve and artery, while at least 11 miRNAs had no mean expression for one or more tissues. Among the results, hsa-mir-567 was not expressed in the artery, cornea, heart, nerve, or vein.

**Figure 3:**
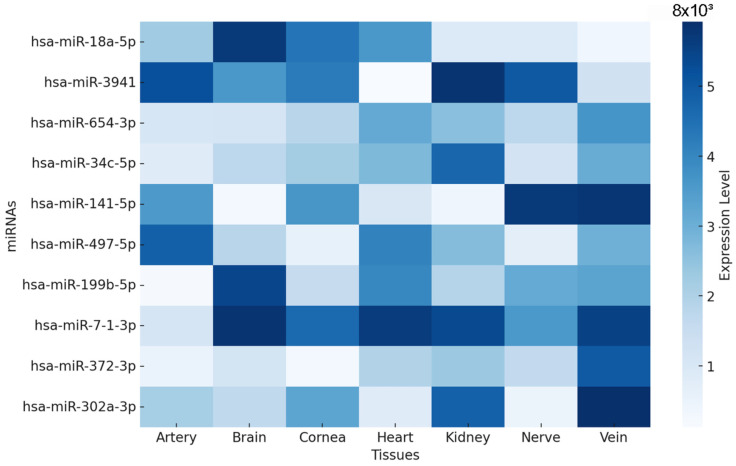
Heatmap of tissue-specific expression profiles from the top 10 predicted miRNAs targeting VEGFA.

The top 10 miRNAs with high means (Figure 3) across tissues demonstrated that these miRNAs exhibited distinct tissue-specific expression patterns. For instance, hsa-miR-302a-3p showed a strikingly higher expression in the cornea, while hsa-miR-3941 and hsa-miR-34c-5p were predominantly expressed in nerve tissue. Conversely, hsa-miR-18a-5p and hsa-miR-654-3p presented a more uniform expression across multiple tissues.

### 3.3. miRNA SNPs Associated with the VEGFA Loss-of-Function Effect

From the top 10 miRNAs predicted to be expressed in diabetic complication-like tissues, only 5 presented an association with the SNPs’ effect of loss in VEGFA regulation (Table 1), while hsa-miR-18a-5p, hsa-miR-3941, hsa-miR-141-5p, hsa-miR-497-5p, and hsa-miR-7-1-3p did not present miRNAs in the loss of regulation of VEGFA. Only hsa-miR-34c-5p presented two SNPs as results.

**Figure 4:**
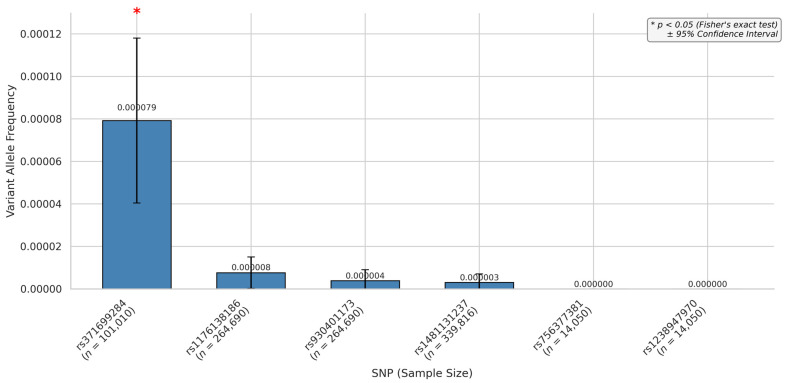
miRNA hsa-miR-654-3p variant frequencies through global population.

The global allele frequency analysis (Figure 4) showed that rs371699284 had the highest frequency among the SNPs, with a value of 0.00079, while other variants exhibited lower frequencies in the global population. However, variants rs1238947970 and rs756377381 did not have any global frequency described.

The rs371699284 distribution across the continents, based on different population screening datasets, demonstrated that the frequency varied according to the dataset investigated. All the studies agreed on a low appearance of the alternative allele, which was very minimally represented overall. A comparison between the alternative allele and the reference showed statistically significant results when evaluated against global data for the datasets from only gnomAD exosomes and gnomAD genomes.

Sample sizes were very high in European populations across all the datasets, with striking differences when compared to other populations. The European subgroup included 203,122 individuals in gnomAD v4—exomes, 78,610 in gnomAD v4—genomes, and 23,452 in ExAC. In contrast, some subgroups had substantially lower representation, such as the South Asian population in the Allele Frequency Aggregator (*n* = 98), Latin American 1 (*n* = 146), and Middle Eastern population in gnomAD v4 genomes (*n* = 314). This variation in sample sizes ranged from over 200,000 individuals to fewer than 100, depending on the dataset and population (Table S5).

**Figure 5:**
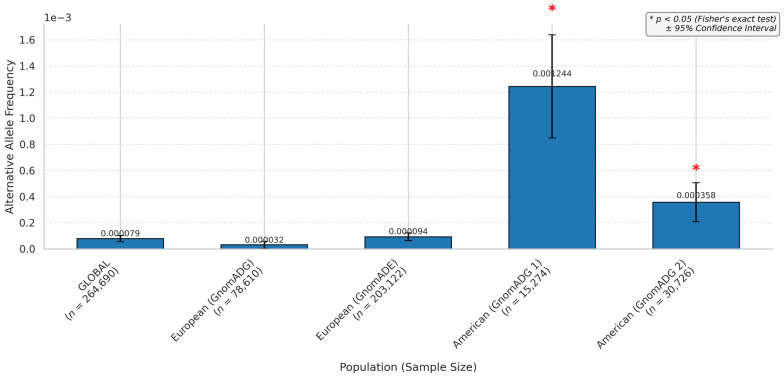
miRNA hsa-miR-654-3p variant frequencies through continents.

The evaluation of the frequency demonstrated statistical significance only for the American and European continents regarding the variant rs371699284 (Figure 5). Global variants also did not show significance. Most dataset studies did not present data for the variant; continents like Africa, America, and Asia had little representation in most datasets (Table S6). The alternative allele representativity for America was observed only in the gnomAD genomes and the exome. In contrast, Europe had representativity across all datasets.

### 3.4. miRNA–mRNA Interaction

**Figure 6:**
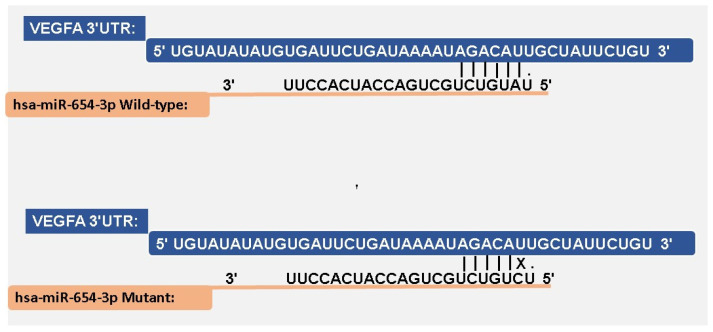
miRNA hsa-miR-654-3p sequence comparisons between the mutant and wild-type.

The comparative alignment between the hsa-miR-654-3p wild-type and mutated hsa-miR-654-3p sequences (Figure 6) showed a single-nucleotide variation, with the U changing to a C. In the wild-type sequence, a base pairing was observed along the 3′UTR seed region with six nucleotides, while in the mutant sequence, it was impaired by the nucleotide change.

Predictions from miRmap (Table 2) at the same target site revealed a ΔG binding free energy of −26.6 kcal/mol for the wild-type miRNA. In contrast, the mutated miRNA showed a reduced binding affinity at the same site, at −19.6 kcal/mol. Additionally, in the mutated sequence, a decrease in the miRmap score, conservation site, and seed match region was observed.

### 3.5. Physiological Implications

**Figure 7:**
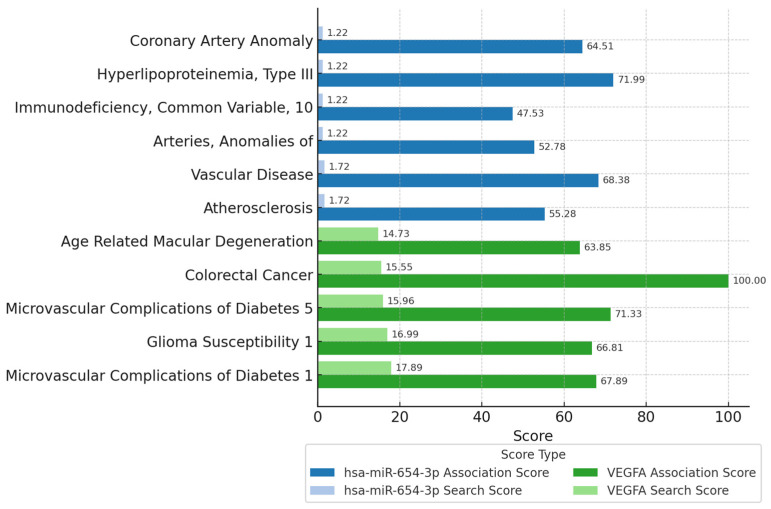
Disease associations of hsa-miR-654-3p and VEGFA with corresponding scores from MalaCards.

Data from the MalaCards (Figure 7) disease algorithm associated hsa-miR-654-3p and VEGFA with several processes, showing higher literature findings in vascular-related disease conditions. miRNA associations were stronger with hyperlipoproteinemia and vascular disease, while VEGFA displayed more promising results in colorectal cancer and micro- and macrovascular conditions. The association score and search score were higher for VEGFA, while for hsa-miR-654-3p, only the association score increased.

## 4. Discussion

The findings of the same miRNAs in prediction and validation evaluations by several tools endorsed the potential of these miRNAs in targeting VEGFA. Additionally, matching results between validation and prediction tools were observed, reflecting shared validated data through the validation and prediction databases and compatibility between in silico and in vivo/in vitro miRNA–mRNA interactions. However, some divergences were found when comparing the results of prediction tools, which can be explained by the distinct algorithmic approaches and predictive criteria [32,33].

The selection of miRNAs based on prediction and experimental validation increased confidence in the potential interactions between miRNAs and mRNAs. Experimental validation provided evidence that these miRNAs interacted with their target genes under physiological conditions. In contrast, predictive tools ensured the recognition of potential binding sites based on sequence complementarity and thermodynamic stability [34,35].

Tissue expression evaluation revealed a lack of some miRNAs in the target tissues, suggesting their potential scarcity of action or biological relevance, even under pathological conditions [36]. The miRNAs with high mean expressions exhibited some tissue variation, emphasizing tissue-specific potential regulatory functions at those biological structures. Certain miRNAs may act as key modulators in specific systems, such as the ocular or nervous tissues, reinforcing their relevance for therapeutic targets or as diagnostic biomarkers [37].

However, the hsa-miR-654-3p exhibited a uniform expression, which suggests a prominent systemic miRNA regulatory effect, indicating that this miRNA affects multiple biological systems beyond a specific tissue, influencing gene expression across them [38].

Identifying SNPs in miRNA regions was challenging; of the 10 miRNAs with prominent tissue expression, only five had SNPs. Highly expressed miRNAs across tissues sometimes showed low data association with known polymorphisms, which may be due to the limitations of sequencing studies. Most studies focused on finding SNPs in protein-coding gene locations, rarely addressing non-coding RNA gene regions [39,40].

The sample representation across continents was unbalanced. It was observed that Europe comprised the vast majority of data. In contrast, the sample sizes from other continents, such as Africa, Latin America, and Asia, were significantly smaller. This divergence undermined the statistical potential of comparative analyses and reduced frequency estimation accuracy, leaving these populations underrepresented. These significant limitations must be considered for interpreting allele distribution [41].

Additionally, the underrepresentation of populations in genomic databases may hinder the discovery of rare or population-specific miRNA variants, reinforcing the need for expansion and more inclusive sequencing efforts. Currently, little data from Africa, Latin America, South Asia, and indigenous groups are available, making these lineages significantly underrepresented in genomic databases [42].

European efforts in sequencing studies are extensive, compared to other continents [43]. However, studies from Asia, especially initiatives in China, are emerging in the landscape of whole-genome evaluations [44,45]. While equity between continents remains lacking, it is believed that approximately 86% of all studies involving whole-genome-wide association studies (GWAS) are from Europe, while ~7% are from Asia, ~1% from Africa, and ~1% from Hispanic/Latino populations [46].

Although European studies have sizeable samples, they represent only a portion of the population, prioritizing primarily Western European countries. Eastern countries like Ukraine and Russia are emerging in this scenario, but it is still insufficient to represent the genomic diversity present in these regions [47].

Linkage disequilibrium is a non-random association of alleles at different loci, where nearby variants are often inherited. This mechanism can influence SNP evaluation; this association may impair linked variants rather than direct functional effects [48]. Although our study did not directly evaluate linkage disequilibrium, we acknowledge its potential impact and now include this limitation in the discussion. Future analyses incorporating this approach can contribute to miRNA-related SNP role interpretation.

Only the rs371699284 allele had a prominent frequency, while the others exhibited low or no frequency. High or low frequencies may indicate potential involvement in common biological pathways. However, low-frequency or population-specific variants could be linked to rare diseases or unique genetic disorders. Recognizing these SNPs can reveal targets for further investigation in precision medicine and studies on disease susceptibility [49,50].

The rs371699284 frequency worldwide demonstrates an association with the European continent. The lower recognition of this variant outside Europe may reflect that this SNP is prevalent in that population. However, the absence of studies from other continents can lead to the misinterpretation of variant distribution due to the scarcity of research. The abundance of sequencing initiatives in Europe allows for a higher recognition of population variation patterns [43].

The miRNA–mRNA interaction showed that rs371699284 (mutant variation) had impaired complementarity compared to the wild-type variant, resulting in a mismatch. This effect weakens binding, potentially reducing the miRNA variant’s interaction with VEGFA. Additionally, binding energy parameters demonstrated favorable interaction values for the wild-type variant. SNPs can weaken the interaction, potentially impairing the miRNA’s regulatory efficiency on the target mRNA [51,52]. Alterations in the target site or miRNA sequence can compromise the thermodynamic stability of the miRNA–mRNA complex, thereby diminishing the silencing effect [53].

Molecular docking studies provide structural interpretations of miRNA–mRNA interactions and can predict how variants like rs371699284 may alter binding affinity and spatial orientation. Simulated interactions between miRNAs, target mRNAs, and AGO proteins help visualize biologically plausible complexes within the cell [54,55]. Prior studies employed Argonaute-assisted docking to model gene regulation mechanisms and miRNA target affinities relevant to visualization tools [56,57]. These approaches could enhance future investigations into the regulation of VEGFA by miRNAs under diabetic conditions.

Previous studies suggested that mutations within the miRNA sequence, particularly in the seed region, can impair mRNA–miRNA binding affinity, leading to a loss of gene silencing efficiency. Genetic variants affecting miRNA sequences have been shown to disrupt the miRNA–mRNA interaction, reducing the post-transcriptional regulation of target genes [5].

The interaction between the wild sequence hsa-miR-654-3p and the VEGFA gene is described in studies with positive validation in a HITS-CLIP (high-throughput sequencing of RNA isolated by crosslinking immunoprecipitation) experiment. In this study, the interaction led to the downregulation of VEGFA expression. HITS-CLIP experiments are well known for their accuracy in identifying direct miRNA–mRNA interactions, providing high-confidence evidence of binding sites and regulatory effects at the transcriptome level [58,59].

Data endorse this miRNA’s role in regulating angiogenesis-related genes. It is also commonly associated with other disorders, such as cancer, where it has been implicated in tumor progression and metastasis [60,61,62].

The has-miR-654-3p is recognized for its role in cell survival, a key mechanism for several biological processes, including vascular homeostasis and being part of the migration, proliferation, and invasion of smooth muscle cells through pyroptosis regulation [63].

VEGFA is associated with several vascular disorders, with an emphasis on diabetic conditions. This gene enhances neovascularization and vascular permeability; however, its overexpression can produce deleterious effects when the angiogenesis process is incomplete or unbalanced [64,65]. Such events can be triggered by hypoxic effects resulting from hyperglycemia and insulin resistance [66,67,68]. Additionally, oxidative stress associated with diabetic conditions exacerbates the detrimental effects of VEGFA, leading to abnormal neovascularization and resulting in fragile vessels, leakage, edema, fibrosis, and inflammation [69,70].

Defects in vascularization cause progressive damage throughout the body, affecting organs such as the retina, kidneys, heart, and peripheral nerves [71,72]. The chronic occurrence of these manifestations can lead to diabetic micro- and macrovascular complications, such as retinopathy, nephropathy, cardiopathy, and neuropathy [73].

The hsa-miR-654-3p is associated with vascular disorders, such as atherosclerosis, type II hyperlipoproteinemia, and anomalous arteries, which are often involved in lipid metabolism alterations that can lead to endothelial disturbances [74,75]. Hyperglycemia increases hypoxia, leading to inflammation and oxidative stress, which can promote the development of the disorder [76,77]. Type 2 diabetes mellitus is recognized as a risk factor for vascular disturbances like atherosclerosis [78]. Therefore, the connection between hsa-miR-654-3p and being a vascular regulator suggests potential effects in type 2 diabetes.

**Figure 8:**
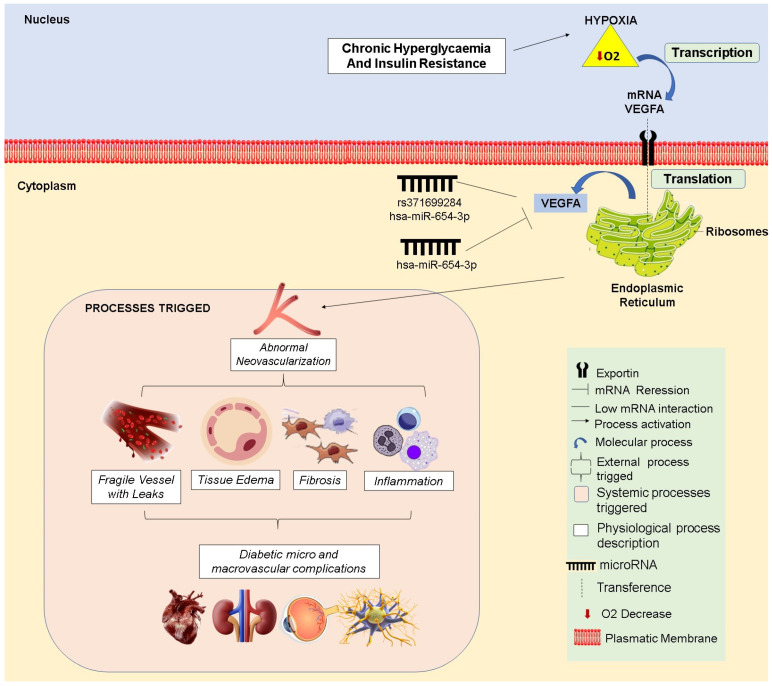
Regulatory impact of wild-type hsa-miR-654-3p and the mutant on VEGFA expression under different conditions.

Furthermore, the regulation of vascularization processes under hyperglycemic and insulin-resistant conditions may involve miRNA hsa-miR-654-3p directly modulating VEGFA expression (Figure 8). By targeting VEGFA, this miRNA may act as a protective factor, preventing its excessive expression commonly seen in individuals affected by metabolic disturbances.

However, the rs371699284 (Figure 8) variation in the miRNA region compromises VEGFA regulation, allowing its expression, which can trigger abnormalities in the vascularization process, as previously described. In this context, we can infer that individuals with these SNPs may be predisposed to VEGFA dysregulation, potentially leading to the development of diabetic complications. Furthermore, these SNPs emerge as potential biomarkers and tools for understanding the complex mechanisms of miRNA-mediated gene regulation [79].

Understanding how such variations affect miRNA binding and target repression can be key to disease progression and opens new therapeutic perspectives [3,80]. Although the present study is based solely on in silico analyses, these findings provide a potential subject for understanding miRNA–VEGFA interactions. Computational predictions have inherent limitations and require experimental biological confirmation [81]. However, in vitro and in vivo studies are necessary to explore these findings and elucidate their functional impact on VEGFA expression and vascular homeostasis.

Our study employed a manually curated text-mining tool that currently provides broader access to structured information on miRNAs and SNPs in public databases and literature [82]. While machine learning approaches hold potential for future applications, particularly in pattern recognition and prediction, existing resources still recognize that text-based strategies are effective for this topic [83,84]. Machine learning-based models for analyzing miRNA–SNP interactions could significantly enhance the analyses and enable the swift interpretation of data.

## 5. Conclusions

This study identified 437 miRNAs associated with VEGFA regulation; 103 were predicted and experimentally validated, with data supporting their biological regulatory potential. Expression analysis of tissue affected by diabetic complications revealed that several of these miRNAs were active, while others exhibited low or no expression profiles. Notably, six highly expressed miRNAs had SNPs that could impair VEGFA silencing. The variant rs371699284 in hsa-miR-654-3p demonstrated reduced binding affinity to VEGFA, suggesting a functional impairment in post-transcriptional regulation.

VEGFA has a well-established role in angiogenesis under hyperglycemic conditions, and given this role, the rs371699284 miRNA variant may contribute to vascular dysregulation and heightened susceptibility to diabetic complications. These findings underscore the importance of integrating genomic variation with functional regulatory analysis to explore potential molecular pathways involved in diabetes-related vascular disorders. Furthermore, these results should be investigated through in vitro and in vivo studies to solidify the SNP’s role.

## Figures and Tables

**Table 1:** miRNAs predicted to regulate VEGFA and their associated SNPs potentially affecting this interaction.

miRNA	SNP
hsa-miR-654-3p	rs371699284
hsa-miR-34c-5p	rs930401173
hsa-miR-34c-5p	rs756377381
hsa-miR-199b-5p	rs1176138186
hsa-miR-372-3p	rs1481131237
hsa-miR-302a-3p	rs1238947970

**Table 2:** miRNA sequences of the wild type and mutant targeting VEGFA 3′UTR interaction results, showing the miRmap score, binding energy (ΔG), site conservation, and seed match type.

miRNA Sequence	miRmap Score	ΔG Binding (kcal/mol)	Conserved Site	Seed Match
Wild Type	−0.412	−23.6	True	8mer
Mutant	−0.295	−19.8	False	7mer-A1

## Data Availability

No new data were created or analyzed in this study. The study is based exclusively on previously published and publicly available datasets. All data sources and database access links are appropriately cited in the manuscript.

## Supplementary Materials

The following supporting information can be downloaded at: https://www.mdpi.com/article/10.3390/biomedicines13051192/s1, Table S1. Predicted miRNAs as a VEGFA regulator though the multimiR tool; Table S2. Correlation between computationally predicted and validated miRNA findings; Table S3. List of predicted and experimentally validated miRNAs targeting the VEGFA gene; Table S4. Tissue-specific expression analysis of the identified miRNAs across different organs; Table S5. Allele frequencies (T>C) across global and subpopulation cohorts from major genomic databases; Table S6. Alternative Allele frequencies (ALT) of the T>C variant in selected populations from gnomAD v4 Exomes and Genomes.
